# Drug Overdose Deaths Among Persons Aged 10–19 Years — United States, July 2019–December 2021

**DOI:** 10.15585/mmwr.mm7150a2

**Published:** 2022-12-16

**Authors:** Lauren J. Tanz, Amanda T. Dinwiddie, Christine L. Mattson, Julie O’Donnell, Nicole L. Davis

**Affiliations:** 1 Division of Overdose Prevention, National Center for Injury Prevention and Control, CDC.

U.S. drug overdose deaths increased 30% from 2019 to 2020 and 15% in 2021, resulting in an estimated 108,000 deaths in 2021.* Among persons aged 14–18 years, overdose deaths increased 94% from 2019 to 2020 and 20% from 2020 to 2021 (*1*), although illicit drug use declined overall among surveyed middle and high school students during 2019–2020 (*2*). Widespread availability of illicitly manufactured fentanyls (IMFs),^†^ proliferation of counterfeit pills resembling prescription drugs but containing IMFs or other illicit drugs,^§^ and ease of purchasing pills through social media^¶^ have increased fatal overdose risk among adolescents (*1*,*3*). Using CDC’s State Unintentional Drug Overdose Reporting System (SUDORS), this report describes trends and characteristics of overdose deaths during July 2019–December 2021 among persons aged 10–19 years (hereafter referred to as adolescents). From July–December 2019 to July–December 2021, median monthly overdose deaths increased 109%, and deaths involving IMFs increased 182%. Approximately 90% of overdose deaths involved opioids, and 83.9% involved IMFs; however, only 35% of decedents had documented opioid use history. Counterfeit pill evidence was present in 24.5% of overdose deaths, and 40.9% of decedents had evidence of mental health conditions or treatment. To prevent overdose deaths among adolescents, urgent efforts are needed, including preventing substance use initiation, strengthening partnerships between public health and public safety to reduce availability of illicit drugs, expanding efforts focused on resilience and connectedness of adolescents to prevent substance misuse and related harms, increasing education regarding IMFs and counterfeit pills, expanding naloxone training and access, and ensuring access to treatment for substance use and mental health disorders.

Funded jurisdictions entered data from death certificates, postmortem toxicology testing, and medical examiner or coroner reports into SUDORS for both unintentional and undetermined intent drug overdose deaths. Monthly trends in all overdose deaths and deaths involving IMFs** (*4*) among decedents aged 10–19 years during July 1, 2019–December 31, 2021 and percent change in the median number of monthly deaths, comparing subsequent 6-month periods, were calculated among 32 jurisdictions.^††^ Percentages of overdose deaths were calculated by demographic characteristics and drugs involved in 47 jurisdictions,^§§^ and by circumstances in 43 jurisdictions,^¶¶^ overall and for decedents within two age groups: 10–14 years and 15–19 years. Analyses were performed using SAS (version 9.4; SAS Institute). This activity was reviewed by CDC and conducted consistent with applicable federal law and CDC policy.***

During July 2019–December 2021, a total of 1,808 adolescent overdose deaths occurred in 32 jurisdictions with available trend data. The number of monthly overdose deaths increased 65% overall, from 31 in July 2019 to 51 in December 2021, peaking at 87 in May 2021 (Figure 1). The number of deaths involving IMFs more than doubled, from 21 to 44 during this period, peaking at 78 in May and August 2021. Median monthly overdose deaths among adolescents increased 109%, from 32.5 during July–December 2019 to 68 during July–December 2021; during the same period, deaths involving IMFs increased 182%, from 22 to 62. Median monthly deaths increased during each 6-month period from July–December 2019 through January–June 2021 and decreased during July–December 2021 but remained approximately twice as high as during July–December 2019.

**FIGURE 1:**
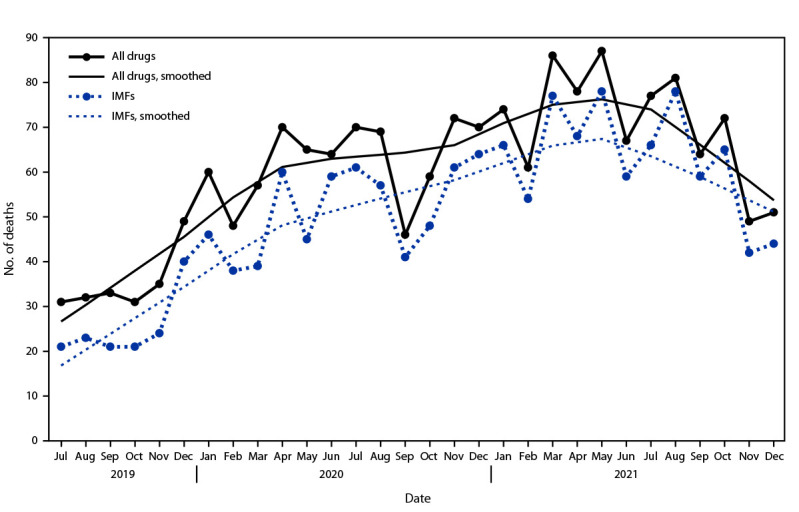
Number of drug overdose deaths and deaths involving* illicitly manufactured fentanyls^†^ among persons aged 10–19 years (N = 1,808), by month — State Unintentional Drug Overdose Reporting System, 32 jurisdictions,^§^ July 2019–December 2021^¶^ **Abbreviations:** IMF = illicitly manufactured fentanyl; SUDORS = State Unintentional Drug Overdose Reporting System. * A drug was considered involved if it was listed as a cause of death on the death certificate or medical examiner or coroner report. ^†^ Includes IMF and fentanyl analogs, which were identified using both toxicology and scene evidence because toxicology alone cannot distinguish between pharmaceutical fentanyl and IMFs. ^§^ Alaska, Arizona, Colorado, Connecticut, Delaware, District of Columbia, Georgia, Illinois, Kansas, Kentucky, Maine, Massachusetts, Minnesota, Missouri, Montana, Nevada, New Hampshire, New Jersey, New Mexico, North Carolina, Ohio, Oklahoma, Oregon, Pennsylvania, Rhode Island, South Dakota, Tennessee, Utah, Vermont, Virginia, Washington, and West Virginia. Illinois, Missouri, and Washington reported deaths from counties that accounted for ≥75% of drug overdose deaths in the state in 2017 per SUDORS funding requirements; all other jurisdictions reported deaths from the full jurisdiction. Jurisdictions reported deaths for all 6-month periods from July 2019 to December 2021. ^¶^ Overdose deaths were smoothed using locally weighted smoothing. The smoothing parameter with the lowest Akaike information criterion was used.

During July 2019–December 2021, among 2,231 adolescent overdose decedents in 47 jurisdictions with available data, more than two thirds (69.0%) were male, and a majority (59.9%) were non-Hispanic White persons (Table). Overall, 2,037 (91.3%) deaths involved at least one opioid; 1,871 (83.9%) involved IMFs, and 1,313 (58.9%) involved IMFs with no other opioids or stimulants. Approximately 10% of deaths involved prescription opioids, and 24.6% involved stimulants. Ninety-three (4.2%) deaths involved neither opioids nor stimulants.

**TABLE:** Characteristics of drug overdose deaths among persons aged 10–19 years (N = 2,231; 47 jurisdictions*) and circumstances surrounding death (N = 1,871; 43 jurisdictions^†^), by age group — State Unintentional Drug Overdose Reporting System, United States, July 2019–December 2021

Characteristic	Age group, yrs, no. (%)		
	10–14 (n = 89)	15–19 (n = 2,142)	Total (N = 2,231)
**Sex**			
Female	40 (44.9)	652 (30.4)	**692 (31.0)**
Male	49 (55.1)	1,490 (69.6)	**1,539 (69.0)**
**Race and ethnicity** ^§^			
American Indian or Alaska Native, non-Hispanic	4 (4.5)	38 (1.8)	**42 (1.9)**
Asian or other Pacific Islander, non-Hispanic	1 (1.1)	26 (1.2)	**27 (1.2)**
Black or African American, non-Hispanic	26 (29.2)	268 (12.6)	**294 (13.3)**
Hispanic or Latino	12 (13.5)	462 (21.7)	**474 (21.4)**
White, non-Hispanic	43 (48.3)	1,285 (60.4)	**1,328 (59.9)**
Multiple races, non-Hispanic	3 (3.4)	49 (2.3)	**52 (2.3)**
**Drugs involved** ^¶^			
Antidepressants	7 (7.9)	79 (3.7)	**86 (3.9)**
Benzodiazepines	5 (5.6)	324 (15.1)	**329 (14.7)**
Any opioids	71 (79.8)	1,966 (91.8)	**2,037 (91.3)**
Heroin**	5 (5.6)	122 (5.7)	**127 (5.7)**
IMFs††	56 (62.9)	1,815 (84.7)	**1,871 (83.9)**
Prescription opioids^§§^	15 (16.9)	202 (9.4)	**217 (9.7)**
Any stimulants	11 (12.4)	537 (25.1)	**548 (24.6)**
Cocaine	4 (4.5)	243 (11.3)	**247 (11.1)**
Methamphetamine	4 (4.5)	255 (11.9)	**259 (11.6)**
**Combinations of opioids and stimulants involved**			
IMFs and stimulants^¶¶^	7 (7.9)	410 (19.1)	**417 (18.7)**
IMFs with no other opioids or stimulants^¶¶^	43 (48.3)	1,270 (59.3)	**1,313 (58.9)**
Prescription opioids with no other opioids or stimulants^¶¶^	15 (16.9)	97 (4.5)	**112 (5.0)**
Neither opioids nor stimulants***	14 (15.7)	79 (3.7)	**93 (4.2)**
**No. of decedents with data from coroner or medical examiner reports (43 jurisdictions)^†^**	68	1,803	**1,871**
**Evidence of overdose circumstances**			
Overdosed at home^§^	45 (66.2)	1,045 (60.2)	**1,090 (60.4)**
Overdosed in house or apartment, not own home^§^	13 (19.1)	378 (21.8)	**391 (21.7)**
Potential bystander present^†††^	54 (79.4)	1,198 (66.4)	**1,252 (66.9)**
No documented overdose response by bystander^§§§^	35 (64.8)	814 (67.9)	**849 (67.8)**
Drug use witnessed	13 (19.1)	357 (19.8)	**370 (19.8)**
Naloxone administered^§^	20 (29.9)	543 (30.4)	**563 (30.3)**
Documentation of no pulse at first responder arrival^§^	38 (55.9)	1,051 (59.5)	**1,089 (59.4)**
**Route of drug use** ^¶¶¶^			
Ingestion	19 (27.9)	427 (23.7)	**446 (23.8)**
Injection	0 (—)	146 (8.1)	**146 (7.8)**
Smoking	12 (17.6)	428 (23.7)	**440 (23.5)**
Snorting	10 (14.7)	421 (23.3)	**431 (23.0)**
No reported route of drug use	38 (55.9)	789 (43.8)	**827 (44.2)**
Evidence of counterfeit pills****	8 (11.8)	451 (25.0)	**459 (24.5)**
**Evidence of drug use history and treatment**			
Alcohol dependence or problem	3 (4.4)	175 (9.7)	**178 (9.5)**
Current treatment for substance use disorders	0 (—)	61 (3.4)	**61 (3.3)**
Ever treated for substance use disorders	1 (1.5)	203 (11.3)	**204 (10.9)**
History of opioid use	9 (13.2)	646 (35.8)	**655 (35.0)**
Previous nonfatal overdose	1 (1.5)	263 (14.6)	**264 (14.1)**

**Abbreviations:** IMF = illicitly manufactured fentanyl; SUDORS = State Unintentional Drug Overdose Reporting System.

* Alabama, Alaska, Arizona, Arkansas, Colorado, Connecticut, Delaware, District of Columbia, Florida, Georgia, Hawaii, Idaho, Illinois, Indiana, Iowa, Kansas, Kentucky, Louisiana, Maine, Maryland, Massachusetts, Michigan, Minnesota, Mississippi, Missouri, Montana, Nebraska, Nevada, New Hampshire, New Jersey, New Mexico, New York, North Carolina, Ohio, Oklahoma, Oregon, Pennsylvania, Rhode Island, South Carolina, South Dakota, Tennessee, Utah, Vermont, Virginia, Washington, West Virginia, and Wisconsin. Alabama, Arkansas, Florida, Hawaii, Illinois, Indiana, Louisiana, Missouri, New York, and Washington reported deaths from counties that accounted for ≥75% of drug overdose deaths in the state in 2017, per SUDORS funding requirements; all other jurisdictions reported deaths from the full jurisdiction. Jurisdictions were included if data were available for at least one 6-month period (July–December 2019, January–June 2020, July–December 2020, January–June 2021, or July–December 2021).

^†^ Alaska, Arizona, Arkansas, Colorado, Connecticut, Delaware, District of Columbia, Florida, Georgia, Hawaii, Illinois, Indiana, Iowa, Kansas, Kentucky, Louisiana, Maine, Maryland, Massachusetts, Michigan, Minnesota, Mississippi, Missouri, Montana, Nebraska, Nevada, New Hampshire, New Jersey, New Mexico, North Carolina, Ohio, Oklahoma, Oregon, Pennsylvania, Rhode Island, South Dakota, Tennessee, Utah, Vermont, Virginia, Washington, West Virginia, and Wisconsin. Arkansas, Florida, Hawaii, Illinois, Indiana, Louisiana, Missouri, and Washington reported deaths from counties that accounted for ≥75% of drug overdose deaths in the state in 2017, per SUDORS funding requirements; all other jurisdictions reported deaths from the full jurisdiction. Jurisdictions were included if data were available for at least one 6-month period (July–December 2019, January–June 2020, July–December 2020, January–June 2021, or July–December 2021) and coroner and medical examiner reports were available for ≥75% of deaths in the included period or periods. Analysis was restricted to decedents with an available coroner or medical examiner report.

^§^ Missing values were excluded from calculations of percentages. Percentages might not sum to 100% because of rounding.

^¶^ A drug was considered involved if it was listed as a cause of death on the death certificate or in the medical examiner or coroner report. Percentages sum to >100% because drug categories are not mutually exclusive.

** Drug entries coded as heroin were heroin and 6-acetylmorphine. In addition, morphine was coded as heroin if detected along with 6-acetylmorphine or if scene, toxicology, or witness evidence indicated presence of known heroin adulterants or impurities (including quinine, procaine, xylazine, noscapine, papaverine, thebaine, or acetylcodeine), injection, illicit drug use, or a history of heroin use.

^††^ Includes IMF and fentanyl analogs, which were identified using both toxicology and scene evidence because toxicology alone cannot distinguish between pharmaceutical fentanyl and IMFs.

^§§^ Drug entries coded as prescription opioids were alfentanil, buprenorphine, butorphanol, codeine, dextrorphan, dihydrocodeine, hydrocodone, hydromorphone, levorphanol, loperamide, meperidine, methadone, morphine, nalbuphine, noscapine, oxycodone, oxymorphone, pentazocine, prescription fentanyl, propoxyphene, sufentanil, tapentadol, and tramadol. Also included as prescription opioids were brand names and metabolites (e.g., nortramadol) of these drugs and combinations of these drugs and nonopioids (e.g., acetaminophen-oxycodone). Morphine was included as prescription only if scene or witness evidence did not indicate likely heroin use and if 6-acetylmorphine was not also detected. Fentanyl was coded as a prescription opioid based on scene, toxicology, or witness evidence.

^¶¶^ Deaths could have involved drugs other than opioids and stimulants (e.g., benzodiazepines).

*** Primarily includes deaths involving antidepressants, antihistamines, and benzodiazepines.

^†††^ For SUDORS, a potential bystander is defined as a person aged ≥11 years who was physically nearby either during or shortly preceding a drug overdose and potentially had an opportunity to intervene or respond to the overdose. This includes any persons in the same structure (e.g., same room or same building, but different room) as the decedent during that time; a family member who was in another room during the fatal incident would be considered a potential bystander if they might have had an opportunity to provide lifesaving measures [e.g., naloxone administration], if adequate resources were available, and if they were aware that an overdose event could occur. Persons in different self-contained parts of larger buildings (e.g., a different apartment in the same apartment building) would not be considered potential bystanders.

^§§§^ Percentages are calculated among decedents with a potential bystander present.

^¶¶¶^ Evidence of injection, smoking, snorting, and ingestion are not mutually exclusive; a death could have evidence of more than one of these routes.

**** Evidence of counterfeit pills included evidence that potential counterfeit pills were found at the scene of the fatal overdose or were consumed by the decedent (according to witness report). Evidence consistent with counterfeit pills included unmarked pills; pills marked with M30 or otherwise appearing like oxycodone pills, with no oxycodone detected by postmortem toxicology testing; pills appearing like alprazolam pills, with no alprazolam detected; pills noted to be counterfeit or potentially counterfeit in the medical examiner or coroner report; pills noted to have contained fentanyl or tested positive for fentanyl; and evidence that the decedent ingested pills with no legitimate pharmaceutical drugs that come in pill form detected by postmortem toxicology testing. Detail regarding potential counterfeit pills in medical examiner or coroner reports varies widely, and certain evidence was likely included in error and certain evidence missed. Counterfeit pills could also possibly be on scene but not consumed by the decedent.

Among 1,871 overdose deaths in 43 jurisdictions with available data on circumstances, 1,090 (60.4%) occurred at the decedent’s home. Potential bystanders^†††^ were present in 1,252 (66.9%) deaths, and 1,089 (59.4%) decedents had no pulse when first responders arrived. Among deaths with one or more potential bystanders present, no documented bystander response was reported for 849 (67.8%), primarily because of spatial separation from decedents (52.9%) and lack of awareness that decedents were using drugs (22.4%). Naloxone administration was documented in 563 (30.3%) deaths. Approximately one quarter of deaths had documentation of ingestion (23.8%), smoking (23.5%), and snorting (23.0%); evidence of injection was documented in 7.8% of deaths. Evidence of counterfeit pills was documented in 24.5% of adolescent deaths. Thirty-five percent of adolescent decedents had documented opioid use history, and 14.1% had evidence of a previous overdose; 10.9% had evidence of substance use disorder treatment, and 3.3% had evidence of current treatment. Approximately 41% of decedents had documented mental health history, including mental health treatment (23.8%), diagnosed depression (19.1%), or suicidal or self-harm behaviors (14.8%) (Figure 2).

**FIGURE 2:**
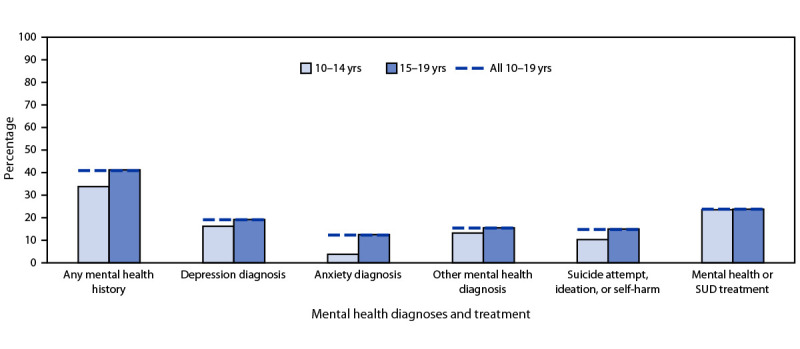
Mental health conditions and treatment history of drug overdose decedents aged 10–19 years (N = 1,871), overall and by age group — State Unintentional Drug Overdose Reporting System, 43 jurisdictions,* July 2019–December 2021^†^^,^^§^ **Abbreviations:** SUD = substance use disorders; SUDORS = State Unintentional Drug Overdose Reporting System. * Alaska, Arizona, Arkansas, Colorado, Connecticut, Delaware, District of Columbia, Florida, Georgia, Hawaii, Illinois, Indiana, Iowa, Kansas, Kentucky, Louisiana, Maine, Maryland, Massachusetts, Michigan, Minnesota, Mississippi, Missouri, Montana, Nebraska, Nevada, New Hampshire, New Jersey, New Mexico, North Carolina, Ohio, Oklahoma, Oregon, Pennsylvania, Rhode Island, South Dakota, Tennessee, Utah, Vermont, Virginia, Washington, West Virginia, and Wisconsin. Arkansas, Florida, Hawaii, Illinois, Indiana, Louisiana, Missouri, and Washington reported deaths from counties that accounted for ≥75% of drug overdose deaths in the state in 2017, per SUDORS funding requirements; all other jurisdictions reported deaths from the full jurisdiction. Jurisdictions were included if data were available for at least one 6-month period (July–December 2019, January–June 2020, July–December 2020, January–June 2021, or July–December 2021) and coroner and medical examiner reports were available for ≥75% of deaths in the included period or periods. Analysis was restricted to decedents with an available coroner and medical examiner report. ^†^ Any mental health history includes at least one of the following: depression diagnosis; anxiety diagnosis; other mental health diagnosis; suicide attempt, ideation, or self-harm; or mental health or SUD treatment. ^§^ Diagnoses are not mutually exclusive.

## Discussion

This report describes five main findings related to overdose deaths among adolescents (persons aged 10–19 years): 1) deaths have increased substantially since the end of 2019; 2) a majority of deaths involved IMFs; 3) nearly one quarter of deaths included evidence of counterfeit pills; 4) two thirds of decedents had a potential bystander present, although most provided no overdose response; and 5) approximately 41% of decedents had a history of mental health conditions or treatment. Overdose prevention efforts promoting awareness of dangers of IMFs and aiming to treat underlying mental health and substance use disorders might help reduce adolescent overdose deaths.

From July–December 2019 to July–December 2021, median monthly overdose deaths among adolescents increased 109%. This increase occurred in the context of decreasing illicit drug use among adolescents during 2019–2020, suggesting that more potent drugs rather than increased use accounted for the increase (*2*). In 2021, among the general population, 73% of overdose deaths involved IMFs (*5*); among adolescents, a higher proportion (84%) involved IMFs, nearly all involved an opioid, and approximately 20% involved both IMFs and stimulants. Overdose prevention messaging aimed toward adolescents that highlights the dangers of IMFs and co-use of opioids and stimulants, and the expansion of naloxone access and training, are essential (*6*). Community-based coalitions, in collaboration with public health entities, can work with schools, physicians, youth-serving organizations, faith-based institutions, and the media to emphasize these messages, support naloxone training and access, and address stigma.^§§§^

Approximately 25% of adolescent deaths had evidence of counterfeit pills, which often mimic the appearance of oxycodone or alprazolam but frequently contain IMFs or other illicit drugs.^¶¶¶^ This percentage is likely underestimated because pills found at scenes were rarely noted as having been tested, and identifying pills as counterfeit based on appearance alone is challenging. The proliferation of counterfeit pills is particularly concerning for adolescents given marketing aimed toward this population and the availability of such pills via social media.****^,^^††††^ Whether adolescents intended to take legitimate pharmaceutical medications or were aware pills were counterfeit is unclear. Regardless, messages that highlight the potential presence of illicit drugs in pills and emphasize that pills should only be used if they are prescribed are important to include in prevention materials for adolescents. Local public health and safety officials should consider issuing warnings regarding counterfeit pills and IMFs to schools, and parents and guardians.^§§§§^

Potential bystanders were present during two thirds of overdose deaths among adolescents; a majority of deaths occurred at home, where bystanders were often family or friends. However, bystanders responded infrequently to the overdose because they were spatially separated (e.g., in another room) or were not aware that the decedent used drugs. Although nearly all deaths involved opioids, just 35% of decedents had documented opioid use history, suggesting recent initiation or lack of awareness by family and friends. In addition, 30% of deaths had evidence that naloxone was administered, suggesting that naloxone might not have been administered soon enough or at a sufficient dosage, or its effectiveness was affected by polydrug use. Educating family and friends to recognize warning signs of drug use, effectively respond to overdose, and monitor adolescents exhibiting risk behaviors associated with drug use might improve bystander response and prevent deaths. Educating adolescents on mitigating practices can also be beneficial, including emphasis on not initiating drug use, not using drugs while alone, using fentanyl test strips,^¶¶¶¶^ and having naloxone readily available for rapid use.

Approximately 41% of decedents had evidence of mental health conditions or treatment; mental health conditions are known risk factors for substance use (*7*). Adolescent mental health was declared a national emergency in 2021 by multiple professional organizations,***** and approximately one third of adolescents reported poor mental health during the COVID-19 pandemic (*8*). Coinciding with the pandemic’s onset, overdose deaths among adolescents continued to increase during January–June 2020, possibly related to declining mental health. Known mental health conditions represent important opportunities for parents, guardians, clinicians, teachers, or other care providers to prevent initiation or recognize signs of substance use (*7*). Protective factors for substance use in adolescents include family engagement, parent and guardian disapproval of substance use, and school connectedness; promoting these might help prevent overdoses (*7*). In addition, implementing programs to prevent adverse childhood experiences that predispose adolescents to risk for substance use should be considered.^†††††^ Among decedents, substance use disorder treatment was rare. Effective substance use disorder treatments for adolescents include psychosocial treatments, such as family-based and cognitive behavioral therapy (*9*) and medications for opioid use disorder (*10*). Given the potential for co-occurring mental health conditions and substance use disorders, integrated treatment approaches might reduce overdose risk (*9*,*10*).

The findings in this report are subject to at least three limitations. First, analyses included 32 to 47 jurisdictions; results might not be generalizable to the entire United States or to other jurisdictions. Second, toxicology testing might differ over time and across jurisdictions; thus, emerging drugs, including new IMFs, might not have been identified. Finally, circumstances surrounding overdose deaths are likely underascertained because of limited investigative information.

Drug overdose deaths among adolescents increased substantially beginning in late 2019. Although deaths appear to have begun declining in late 2021, they are still alarmingly higher than in 2019. Urgent efforts to prevent overdose deaths among adolescents are needed and include 1) preventing substance use initiation and promoting protective factors; 2) strengthening partnerships between public health and public safety to reduce availability of illicit drugs; 3) expanding efforts focused on resilience and connectedness of adolescents to help prevent substance misuse and related harms; 4) educating about dangers of IMFs and counterfeit pills; 5) promoting safer drug use for those who use drugs, such as not using drugs while alone and having naloxone readily available; 6) expanding naloxone access and training family and friends in overdose recognition and response; and 7) ensuring access to effective, evidence-based substance use disorder and mental health treatment. Collaboration among public health and safety agencies, physicians, mental health and substance use treatment providers, and educators to implement these efforts could save lives.

**Summary**

**What is already known about this topic?**

From 2019 to 2021, overdose deaths among persons aged 14–18 years increased.

**What is added by this report?**

Median monthly overdose deaths among persons aged 10–19 years (adolescents) increased 109% from July–December 2019 to July–December 2021; deaths involving illicitly manufactured fentanyls (IMFs) increased 182%. Approximately 90% of deaths involved opioids and 84% involved IMFs. Counterfeit pills were present in nearly 25% of deaths. Two-thirds of decedents had one or more potential bystanders present, but most provided no overdose response. Approximately 41% of decedents had evidence of mental health conditions or treatment.

**What are the implications for public health practice?**

Educating adolescents about the dangers of IMFs and counterfeit pills, working with public safety to reduce availability of illicit drugs, and ensuring access to evidence-based substance use and mental health treatment could save lives.

## Footnotes

* https://www.cdc.gov/nchs/nvss/vsrr/drug-overdose-data.htm (Accessed November 4, 2022).

†
https://www.dea.gov/sites/default/files/2021-02/DIR-008-21%202020%20National%20Drug%20Threat%20Assessment_WEB.pdf

§
https://www.dea.gov/alert/sharp-increase-fake-prescription-pills-containing-fentanyl-and-meth

¶
https://www.dea.gov/sites/default/files/2022-03/20220208-DEA_Social%20Media%20Drug%20Trafficking%20Threat%20Overview.pdf

** Fentanyl was classified as likely illicitly manufactured using toxicology, scene, and witness evidence. In the absence of sufficient evidence to classify fentanyl as illicit or prescription (16% of deaths involving fentanyl), fentanyl was classified as illicit because the vast majority of fentanyl overdose deaths involve illicit fentanyl. All fentanyl analogs except alfentanil, remifentanil, and sufentanil (which have legitimate human medical use) were included as illicitly manufactured fentanyls.

†† Alaska, Arizona, Colorado, Connecticut, Delaware, District of Columbia, Georgia, Illinois, Kansas, Kentucky, Maine, Massachusetts, Minnesota, Missouri, Montana, Nevada, New Hampshire, New Jersey, New Mexico, North Carolina, Ohio, Oklahoma, Oregon, Pennsylvania, Rhode Island, South Dakota, Tennessee, Utah, Vermont, Virginia, Washington, and West Virginia. Illinois, Missouri, and Washington reported deaths from counties that accounted for ≥75% of drug overdose deaths in the state in 2017, per SUDORS funding requirements; all other jurisdictions reported deaths from the full jurisdiction. Jurisdictions reported deaths for all 6-month periods from July 2019 to December 2021.

§§ Alabama, Alaska, Arizona, Arkansas, Colorado, Connecticut, Delaware, District of Columbia, Florida, Georgia, Hawaii, Idaho, Illinois, Indiana, Iowa, Kansas, Kentucky, Louisiana, Maine, Maryland, Massachusetts, Michigan, Minnesota, Mississippi, Missouri, Montana, Nebraska, Nevada, New Hampshire, New Jersey, New Mexico, New York, North Carolina, Ohio, Oklahoma, Oregon, Pennsylvania, Rhode Island, South Carolina, South Dakota, Tennessee, Utah, Vermont, Virginia, Washington, West Virginia, and Wisconsin. Alabama, Arkansas, Florida, Hawaii, Illinois, Indiana, Louisiana, Missouri, New York, and Washington reported deaths from counties that accounted for ≥75% of drug overdose deaths in the state in 2017, per SUDORS funding requirements; all other jurisdictions reported deaths from the full jurisdiction. Jurisdictions were included if data were available for at least one 6-month period (July–December 2019, January–June 2020, July–December 2020, January–June 2021, or July–December 2021).

¶¶ Alaska, Arizona, Arkansas, Colorado, Connecticut, Delaware, District of Columbia, Florida, Georgia, Hawaii, Illinois, Indiana, Iowa, Kansas, Kentucky, Louisiana, Maine, Maryland, Massachusetts, Michigan, Minnesota, Mississippi, Missouri, Montana, Nebraska, Nevada, New Hampshire, New Jersey, New Mexico, North Carolina, Ohio, Oklahoma, Oregon, Pennsylvania, Rhode Island, South Dakota, Tennessee, Utah, Vermont, Virginia, Washington, West Virginia, and Wisconsin. Arkansas, Florida, Hawaii, Illinois, Indiana, Louisiana, Missouri, and Washington reported deaths from counties that accounted for ≥75% of drug overdose deaths in the state in 2017, per SUDORS funding requirements; all other jurisdictions reported deaths from the full jurisdiction. Jurisdictions were included if data were available for at least one 6-month period (July–December 2019, January–June 2020, July–December 2020, January–June 2021, or July–December 2021), and coroner and medical examiner reports were available for ≥75% of deaths in the included period or periods. Analysis was restricted to decedents with an available coroner or medical examiner report.

*** 45 C.F.R. part 46.102(l)(2), 21 C.F.R. part 56; 42 U.S.C. Sect. 241(d); 5 U.S.C. Sect. 552a; 44 U.S.C. Sect. 3501 et seq.

††† For SUDORS, a potential bystander is defined as a person aged ≥11 years who was physically nearby either during or shortly preceding a drug overdose and potentially had an opportunity to intervene or respond to the overdose. This includes any persons in the same structure (e.g., same room or same building, but different room) as the decedent during that time; a family member who was in another room during the fatal incident would be considered a potential bystander if they might have had an opportunity to provide lifesaving measures [e.g., naloxone administration], if adequate resources were available, and if they were aware that an overdose event could occur. Persons in different self-contained parts of larger buildings (e.g., a different apartment in the same apartment building) would not be considered potential bystanders.

§§§ https://www.cdc.gov/drugoverdose/featured-topics/drug-free-communities.html (Accessed November 4, 2022).

¶¶¶ https://www.dea.gov/alert/sharp-increase-fake-prescription-pills-containing-fentanyl-and-meth (Accessed November 4, 2022).

****
https://www.dea.gov/sites/default/files/2022-03/20220208-DEA_Social%20Media%20Drug%20Trafficking%20Threat%20Overview.pdf

††††
https://www.dea.gov/stories/2021/2021-07/2021-07-23/counterfeit-drugs-social-media

§§§§
https://www.dea.gov/sites/default/files/2022-09/DEA-OPCK_Parent%20flyer_V6.pdf

¶¶¶¶
https://www.cdc.gov/stopoverdose/fentanyl/fentanyl-test-strips.html

***** https://www.aap.org/en/advocacy/child-and-adolescent-healthy-mental-development/aap-aacap-cha-declaration-of-a-national-emergency-in-child-and-adolescent-mental-health/?_ga = 2.148070580.226118470.1666026568-1362783567.1666026568 (Accessed November 4, 2022).

†††††
https://www.cdc.gov/violenceprevention/pdf/preventingACES.pdf
